# Comparison of Linear Poly Ethylene Imine (LPEI) and Poly L-Lysine (PLL) in Fabrication of CHOK_1_ Cell-Loaded Multilayer Alginate Microcapsules

**DOI:** 10.34172/apb.2020.035

**Published:** 2020-02-18

**Authors:** Fariba Hajifathaliha, Arash Mahboubi, Elham Mohit, Noushin Bolourchian, Vahid Khalaj, Leila Nematollahi

**Affiliations:** ^1^Food Safety Research Center, Shahid Beheshti University of Medical Sciences, Tehran, Iran.; ^2^Student Research Committee, Department of Pharmaceutics, School of Pharmacy, Shahid Beheshti University of Medical Sciences, Tehran, Iran.; ^3^Department of Pharmaceutical Biotechnology, School of Pharmacy, Shahid Beheshti University of Medical Sciences, Tehran, Iran.; ^4^Biotechnology Research Center, Pasteur Institute of Iran, Tehran, Iran.

**Keywords:** Alginic acid, Cell microencapsulation, CHOK_1_, Poly ethylene imine, Poly l-lysine

## Abstract

***Purpose:*** Poly l-lysine (PLL) has been introduced as a strengthening covering layer for alginate microcapsules which are the most convenient way for cell encapsulation. Some disadvantages of PLL such as high price and low biocompatibility have prompted scientists to find better alternatives. Linear poly ethylene imine (LPEI), thanks to its highly similar structure to PLL, could be considered as a proper cost-effective alternative. In this study LPEI and PLL were compared as covering layers of cell-loaded alginate-LPEI-alginate (cALA) and alginate-PLL-alginate (cAPA) microcapsules.

***Methods:*** In addition to the physico-mechanical properties, the encapsulation efficiency, cell survival post encapsulation, cell viability, and cellular metabolic activity within the microcapsules were evaluated using trypan blue, live/dead cell staining, and MTT test, respectively.

***Results:*** Physico-mechanical evaluation of the microcapsules revealed that the cell microencapsulation process did not affect their shape, size, and mechanical stability. Although the encapsulation efficiency for cALA and cAPA was not different (*P* >0.05), cell survival post encapsulation was higher in cALA than in cAPA (*P*<0.05) which could be the reason for the higher cell viability and also cellular metabolic activity within these microcapsules in comparison to cAPA.

***Conclusion:*** Here, based on these results, ALA could be introduced as a preferable alternative to APA for cell encapsulation.

## Introduction

Cell microencapsulation has been used as a multifunctional technology in treatment of different chronic disorders such as diabetes mellitus, central nervous system or cardiovascular problems, fabrication of bioreactors to produce monoclonal antibodies, etc.^1,2^ Lim and Sun are the inventors of cell microencapsulation technology. They used alginate as a semipermeable membrane to enclose live islets.^3^

Multilayer alginate microcapsules are the most common devices in cell encapsulation. This method is performed by immobilization of live cells into the semipermeable anionic alginate microcapsules which are covered by poly electrolyte complexes. The first coating layer (cationic layer) is used to restrict the pore sizes and enhance the mechanical stability of the microcapsules, while the final anionic layer is added to cover the excess surface positive charges, leading to decrease microcapsules cytotoxicity.^4-7^ Poly l-lysine (PLL), as the covering layer of alginate microcapsules, is the oldest and the most utilized cationic polymer. However, because of some disadvantages such as its high price, low biocompatibility, etc in many studies, introducing a proper alternative to PLL is desirable. Linear poly ethylene imine (LPEI), a more cost-effective polymer with a great similarity to PLL, could be recommended as a proper alternative.

In our previous study, to ensure the similar performance of LPEI and PLL, the physical properties, surface morphology, mechanical strength, cytotoxicity, and long-term stability of ALA and APA microcapsules were investigated.^8^ Since the ALA microcapsules showed similar results to APA in all the physico-mechanical and cytotoxicity evaluations, it could be employed in further comparative investigations. The purpose of this study was to finalize the comparison between PLL and LPEI as well as evaluation of these two polymers for the encapsulation of CHOK1; the cell line which has attracted a great deal of attention in manufacturing of biopharmaceuticals.^9^ In this study, cell-loaded alginate-LPEI-alginate (cALA) and alginate-PLL-alginate (cAPA) were fabricated using electrostatic bead generator. The shape, size, and mechanical stability have been investigated in addition to the encapsulation efficiency, cell survival post encapsulation, cell viability, and cellular metabolic activity inside the cALA and cAPA microcapsules.

## Materials and Methods

### Materials

Adherent CHOK1 cell line (ATCC CRL-9661) was granted by Pasteur Institute of Iran. Ham’s F12 nutrient mixture was obtained from Inoclon Co. (Iran). Antibiotic solution of Penicillin/Streptomycin, and trypsin 10X were obtained from Biosera Co. (England). Fetal bovine serum was purchased from Gibco Co. (USA). High G content alginic acid (MW, 100 000-200 000, G content, 70%), calcium chloride, linear PEI (Mn~10 000), PLL (MW 70 000-150 000, 0.01%), HEPES, MTT reagent, trypan blue, fluorescein diacetate, propidium iodide, L-glutamine, and DMSO were purchased from Sigma-Aldrich (USA).

#### Cell culture

CHOK_1_ cells were cultured in Ham’s F12 medium composed of 10% fetal bovine serum (FBS), 1% glutamine, 1% antibiotic solution (penicillin/streptomycin) maintained at 37°C in a humidified 5% CO2 atmosphere. The cells were passaged every 2-3 days.

#### CHOK_1_ growth curve

Once the cell-line became confluent, it was counted in a Neubauer chamber at 1:3 dilution with trypan blue (TB) solution. The cells were seeded at a density of 20 000 cells per well in two 12-well plates and counted by TB dye exclusion for 8 days at 24-hour intervals. The medium was changed once every three days. CHOK1 growth curve was obtained by plotting the number of viable cells versus incubation time.

#### Microencapsulation

CHOK1 cells were trypsinized then centrifuged for 5 minutes at 1100 rpm. After removing the supernatant, cell pellet was resuspended in the cell culture medium, where 1 mL of cell suspension was mixed with 4 mL of 2% sterile-filtered sodium alginate solution. The cell concentration in sodium alginate solution was approximately (1.5-2)*10^6^ cell/mL. The microcapsules were produced using electrostatic bead generator under conditions stated elsewhere.^8^ Briefly, using electrostatic bead generator (flow rate: 15 mL/h, applied voltage: 10 kV), 5 mL of cell containing sodium alginate solution was extruded into the 120 mM CaCl_2_ solution. The resultant cell loaded microcapsules were allowed to remain in CaCl_2_ solution for 15 minutes on a magnetic stirrer. They were then filtered and divided into two parts, with each part incubated in 0.03% PLL or 0.03% LPEI solution for 5 minutes. Finally, the microcapsules were incubated in 0.2% sodium alginate solution for 5 minutes. Sodium alginate and the cationic polymer solutions were dissolved in a 0.9% NaCl solution containing 0.24% HEPES (pH 7.2 to 7.4) and sterilized using filter 0.22 µm, while the other solutions were sterilized via autoclaving. Unless otherwise stated, all the concentrations have been shown in percentage of weight to volume (W/V). There was a washing step in 0.9% NaCl solution after incubation in each solution. The cell loaded microcapsules were kept at 37°C in a humidified 5% CO2 atmosphere for further investigations.^10^

#### Characterization of the microcapsules

#### Shape and size

The shape and size of the microcapsules were evaluated by inverted microscope (OPTIKA, XDS-2FL) equipped with a digital camera. In each batch, 30 microcapsules were placed in a plate and observed under the microscope at 40X magnification. The program installed on the camera measured the microcapsules diameter through three points on their perimeter. All the experiments were performed in triplicates with the results reported as mean ± SD.^8,11^

#### Surface morphology: SEM studies

The surface morphology of the microcapsules was studied using scanning electron microscope (SEM). Freeze-dried microcapsules were fixed on conducting stubs and vacuum coated with gold palladium film using a sputter coater (Edward S-150, UK). Images were taken using 26 kV electron beam intensity in a scanning electron microscope (KYKY-EM3200, China).^12^

#### Long-term stability of the microcapsules

To investigate the long-term stability of the microcapsules, cALA and cAPA were transferred to a 6-well plate, with 5 mL of culture medium added to each well and kept at 37˚C in a humidified 5% CO_2_ atmosphere. The medium was changed every three days. The microcapsules stability was investigated according to their size and integrity using inverted microscope. The nature of these microcapsules leads them to absorb water whereby their diameter would increase. Enlargement of microcapsules size is inversely proportional to their stability.^8,13^ The experiments were conducted three times, with the results reported as mean ± SD.

### Encapsulation efficiency

Encapsulation efficiency (EE%) for cALA and cAPA microcapsules was investigated using Maguire et al method with some modifications.^10^ First, a known concentration of cells was encapsulated in ALA and APA microcapsules. Immediately following microencapsulation (as described earlier), the microcapsules were incubated in PBS (pH. 6.8) for 30 minutes on a magnetic stirrer (cALA and cAPA in separate beakers).^14-16^ Under this condition, the microcapsules were depolymerized and the encapsulated cells were released to the medium. Then, this mixture was transferred to a falcon tube and centrifuged for 15 minutes at 2000 rpm. The cells in the pellet were counted using TB dye exclusion. Finally, the EE% was calculated using equation 1. In order to compare the EE% between cALA and cAPA microcapsules, this parameter was calculated three times for each of them separately, where the results were reported as mean ± SD.

EE% = (N1/N0)*100 (1)

N_0_ is the initial number of cells in the alginate solution.

N_1_ represents the number of cells recovered from the depolymerized cALA or cAPA microcapsules.

### Cell survival post encapsulation

To investigate the compatibility of the microencapsulation procedure with CHOK_1_ cells, the live/dead cells ratio inside the microcapsules was investigated one day after the microencapsulation. Fluorescein diacetate (FDA) and propidium iodide (PI) (as will be described later in live/dead cell staining section), were used for double staining of the cells in microcapsules. FDA (non-fluorescent) is taken up by live cells and converted into the green fluorescent metabolite; fluorescein. In contrast, PI cannot pass through a live cell membrane. Passing through the damaged dead cell membranes, it stains the nucleus and intercalates with the DNA double helix of the cell. Red signal under the fluorescent microscope serves as an indicator for dead cells.

### Live/dead cell staining

#### Solutions preparation

Stock solutions of FDA (5 mg/mL) and PI (2 mg/mL) were prepared in acetone and PBS, respectively. These solutions are stable for several months when stored in dark at 2-8°C for PI and at -20°C for FDA.17 A fresh working solution was prepared daily composed of 5 ml PBS, 10 µL FDA (5 mg/mL), and 50 µL PI (2 mg/mL). The concentration of components may need to be adjusted for each cell line.

#### Fluorescent double staining

Cell-loaded microcapsules were incubated in 1 mL of the working solution at room temperature and dark environment for 15 minutes, then washed in PBS. Fluorescent images were taken using a fluorescence microscope (OPTIKA, XDS-2FL) equipped with a 495 nm excitation filter and emission filters of 515 and 635 nm. Green cells were considered alive, while red cells were considered dead. Finally, the images were quantified byMATLAB software.

#### Quantification of fluorescent images

The number of red and green pixels in each picture was calculated using MATLAB, represented by parameters nR and nG. Detection was based on the intensity of the pixel color, with a threshold of 0.1 for both green and red pixels. Note that although dead and living cells are demonstrated in a single merged picture, calculations have been performed on separate pictures for better precision. The ratio of living or dead cells to the total number of cells is calculated as nG/(nG+nR) or nR/(nG+nR), respectively.^18^

### Cell viability within 4 weeks; Fluorescent double staining

To investigate the suitability of the cALA and cAPA microcapsules and overall storage conditions, microcapsules kept at 37˚C in a humidified 5% CO2 atmosphere, were monitored weekly using FDA/PI double staining method under fluorescence microscope.

### Metabolic activity inside the microcapsules; MTT test

The encapsulated cells in ALA and APA were compared for metabolic activity using MTT assay. Briefly, in each 96-wells plate (in total, five 96-wells plates were seeded; 4 plates for separate, weekly assessments and one plate for evaluating metabolic activity at the day of microcapsules fabrication; day 0). Separate columns in each plate were dedicated to cALA, cAPA, and blank medium (composed of culture medium, MTT reagent, and DMSO), and seeded by 150 µL of each relevant material (cALA, cAPA or blank medium). The plates were kept at 37°C, 5% CO_2_ and 97% rh. At each test day, 20 µL of MTT solution (5 mg/mL) was added to each well, then plate covered with aluminum foil and kept in an incubator at 37˚C for 4 hours. After incubation, MTT solution was removed by aspiration; then 100 µL of DMSO was added to each well to ensure the dissolution of the insoluble formazan crystals, and the plate was kept on the shaker for 45 minutes then optical densities were measured at 570 nm in a microplate reader (SYNERGY, BioTek, USA).^19^

### Statistical analysis

The statistical analysis was conducted using SPSS version 17.0. Student’s *t* test was utilized to detect significant differences. *P* value <0.05 was considered statistically significant.

## Results and Discussion

### Physical characteristics

**Figure 1:**
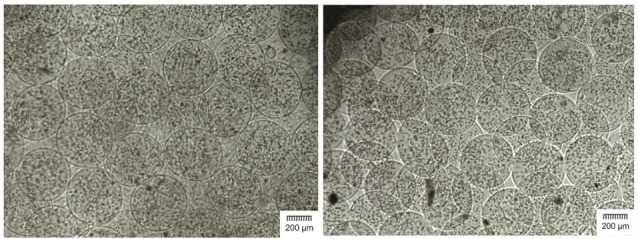
Visual images of cell-loaded alginate-linear poly ethylene imine-alginate (cALA) (left) and cell-loaded alginate-poly l lysine-alginate (cAPA) (right) microcapsules observed at 40X magnification under an inverted light microscope (scale bar is 200 µm).

**Figure 2:**
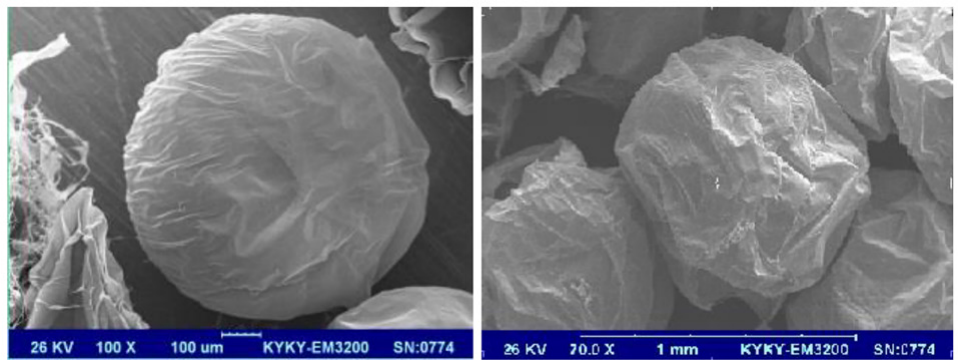
SEM photographs of cell-loaded alginate-linear poly ethylene imine-alginate (cALA) (left) and cell-loaded alginate-poly l lysine-alginate (cAPA) (right) microcapsules.

Evaluation of the microcapsules size in each batch showed that the average diameter of cALA and cAPA microcapsules was 449.29 ± 26.97 and 445.14 ± 25.86 (µm), respectively (n = 3). As stated in the previous study,^8^ the images taken by the inverted microscope along with the SEM images of cALA and cAPA microcapsules clearly indicated that the electrostatic bead generator method along with the influential variable parameters such as the concentration of alginate solution, distance between the needle and the surface of CaCl_2_ solution, extrusion rate, etc. were all well suited for fabrication of microcapsules with a similar size and shape and no marked differences (Figures 1 and 2).

#### Long-term stability of the microcapsules

**Figure 3:**
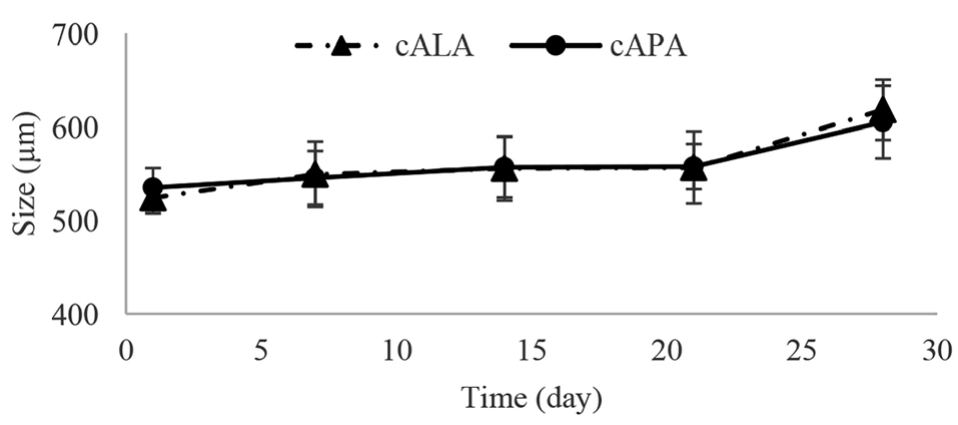
Size increasing patterns for cell-loaded alginate-linear poly ethylene imine-alginate (cALA) and cell-loaded alginate-poly l lysine-alginate (cAPA) microcapsules within 4 weeks, each point represents mean ± SD, (n=3).

The ionic nature of polyelectrolyte complexes would make them vulnerable to the environmental conditions (e.g. culture medium composition), causing instability in their structure. Therefore, stability of the microcapsules during a specified time period should be guaranteed. Figure 3 depicts how the size of microcapsules changed within 4 weeks while being incubated at 37°C, 5% CO2 and 97% rh. According to this figure, since cALA and cAPA microcapsules showed similar patterns of size growth, it could be concluded that they may show similar stability as well. It is worth mentioning that like empty ALA and APA, almost all of the cALA and cAPA microcapsules also remained intact within 4 weeks.^8^ However, there are also studies suggesting opposing results. In the study conducted by Rokstad et al the stability of empty APA microcapsules (standard Ca/Ba) was reported to be far greater than that of cell-loaded microcapsules. They also recorded the highest amount of endostatin secretion from the same type of microcapsules (standard Ca/Ba) which could indicate the high rate of cell growth inside these microcapsules. As a result, these microcapsules could be filled, which might be the reason for their lower stability.^20^ It could be concluded that the stability and lifelong of the microcapsules could be influenced by cell type, and would be enhanced by decreasing the initial number of cells.^21^

#### Encapsulation efficiency

Encapsulation efficiency, as a yield of procedure, should be evaluated regardless of the purpose of the encapsulation or the type of the encapsulated substance. However, despite many studies in the field of cell-encapsulation, this parameter has remained underreported. Encapsulation efficiency is influenced by many factors such as molecular weight and pore size of the materials, which are used to fabricate and cover the microcapsules.^22^ In this study, all the procedures for fabrication of the cALA and cAPA were the same, with the only difference being the type of the cationic polymer; LPEI or PLL. The evaluation of EE% for cALA and cAPA revealed no significant difference between these two cationic polymers in covering the alginate microcapsules (Table 1) (*P>* 0.05). This high yield of encapsulation is also in line with Maguire et al who reported 98% cell recovery for embryonic stem cells encapsulated in alginate-PLL microcapsules.^10^

**Table 1:** EE% for cell-loaded alginate-linear poly ethylene imine-alginate (cALA) and cell-loaded alginate-poly l lysine-alginate (cAPA) microcapsules

**Type of microcapsules**	**Encapsulation efficiency (%)**
cALA	93.98±2.94
cAPA	96.06±1.41

*P* >  0.05 (n=3).

#### Cell survival post encapsulation

**Figure 4:**
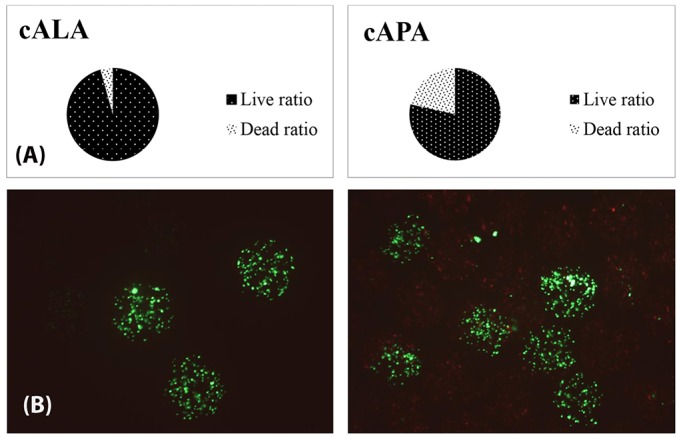
(A) Quantified representation of cell Survival 1 day after microencapsulation, *P* < 0.05, (n=3). (B), Fluorescence microscope images (merged) of encapsulated cells stained with FDA (green) and PI (red), 1 day after microencapsulation, left, cell-loaded alginate-linear poly ethylene imine-alginate (cALA), right, cell-loaded alginate-poly l lysine-alginate (cAPA).

To investigate the cell survival one day after microencapsulation, the cALA and cAPA microcapsules were stained with FDA/PI and imaged, after which the live/dead cells ratio inside them was evaluated. As observed in Figure 4A and 4B, cell survival one day after microencapsulation has been significantly higher for cALA than for cAPA (95.5 ± 0.98 vs 78.48 ± 1.56). This was also proved by MTT test in which, OD values for cALA and cAPA at the day of microencapsulation (day 0) were 0.37 ± 0.06 and 0.1 ± 0.01, respectively (*P* < 0.05). This finding is in agreement with Maguire et al along with Rokstad et al who separately showed the harmfulness of both the encapsulation process and PLL solution to the cell lines.^10,21^ Fischer et al as well as Jeong et al listed molecular weight, charge density, structure, and conformational flexibility of the polymers as the influential parameters in their biocompatibility. As they stated, cytotoxicity of polymers is a function of molecular weight. Therefore, in this study the lower cell survival for cAPA in comparison with cALA could be due to the higher molecular weight of PLL than LPEI.^23,24^

#### Evaluation of cell viability; FDA/PI staining

**Figure 5:**
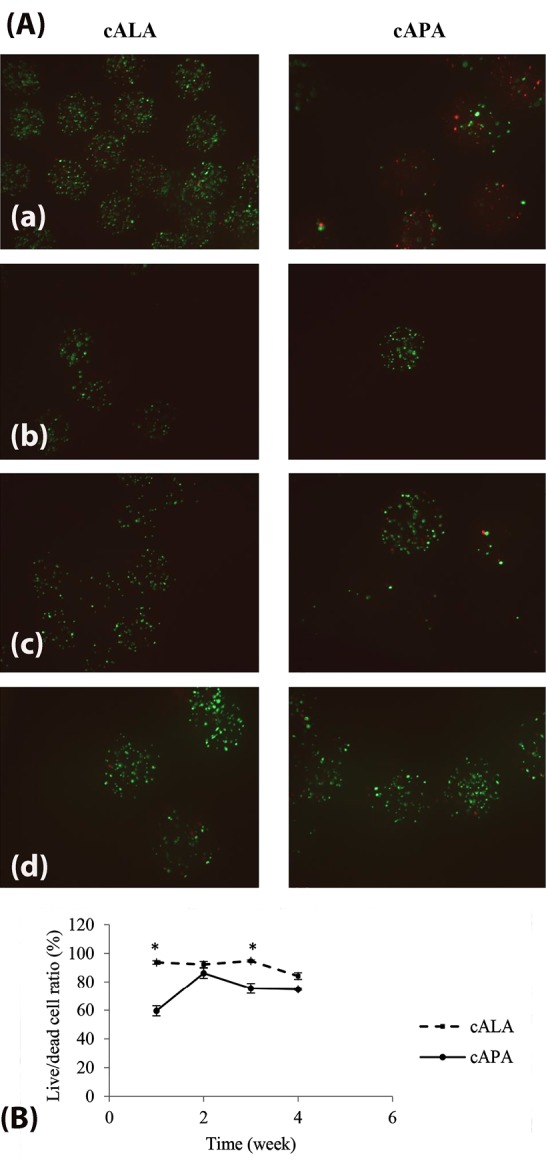
(A) Fluorescence microscope images (merged) of encapsulated cells stained with FDA (green) and PI (red), (a), first week,.(b), second week, (c) third week and (d) fourth week post-encapsulation. (B) Quantitative representation of the live/dead cells ratio within 4 weeks. Results are expressed as mean± SD, (n = 3), *P<0.05, cALA: cell-loaded alginate-linear poly ethylene imine-alginate, cAPA: cell-loaded alginate-poly l lysine-alginate (cAPA).

The main purpose of cell microencapsulation is to maintain them viable. Evaluation of cell viability inside the microcapsules was carried out within four weeks, using live/dead cells staining with FDA and PI (Figure 5A). Figure 5B represents the quantified data obtained from the fluorescent images. As can be seen, the live/dead cells ratio after one week for cALA has been significantly higher than for cAPA (*P* < 0.05). This finding supports the results of section 3.4, where higher post encapsulation cell survival for cALA led to the higher cell viability at the end of the first week. However, the higher rate of cell proliferation in cAPA in comparison with cALA resulted in the closer live/dead cells ratio at the end of the second week (*P* >  0.05). Since living cells would compete for nutrients and O_2_ uptake, the lower number of live cells in cAPA microcapsules could be the reason for the higher proliferation rate in them (within the interval between the first and second week) in comparison with cALA. Ross and Chang came up with a similar result. They introduced the concept of “peak capacity” as the ability of encapsulated cells to grow rapidly in microcapsules to fill them.^25^ It seems that, until the end of the second week, the encapsulated cells in APA have grown rapidly and reached their peak capacity. The live/dead cells ratio gradually declined from the second week to the fourth week. This is in agreement with Kuijlen et al who reported gradual drop in the number of CHOK_1_ live cells after 16 days post encapsulation, due to the increase in their number and competition between them.^26^ However, there are studies reporting different growth patterns inside the microcapsules, mainly based on the type of the encapsulated cell lines.^21,27^

#### Evaluation of metabolic activity; MTT test

**Figure 6:**
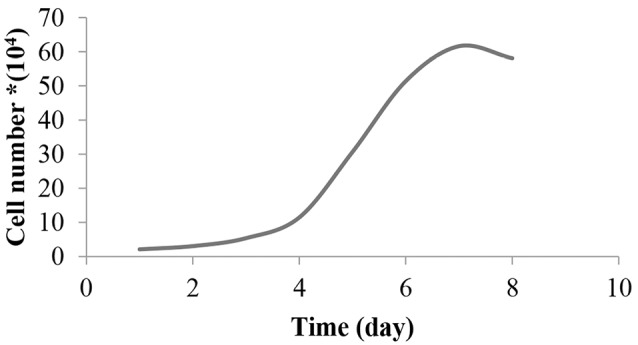
CHOK1 cell growth curve.

**Figure 7:**
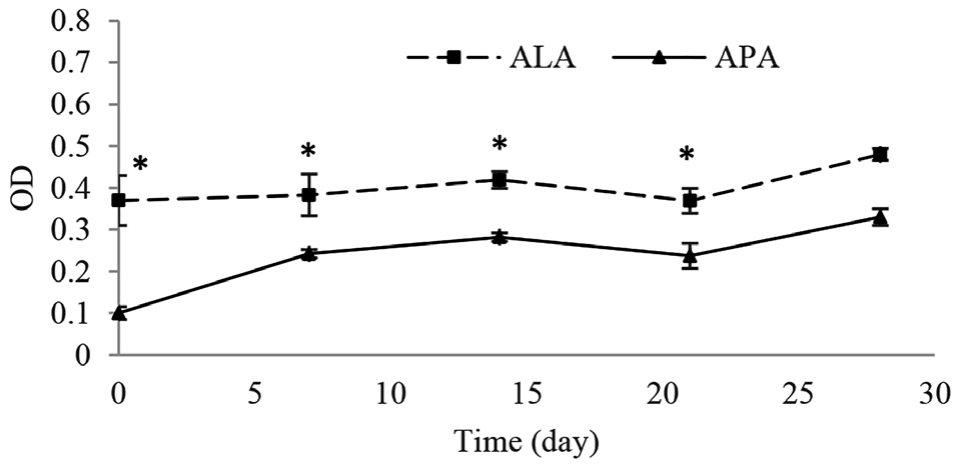
Determination of the metabolic activity of CHOK1 cells immobilized in ALA: alginate-linear poly ethylene imine-alginate and APA: alginate-poly l lysine-alginate microcapsules by MTT test within 4 weeks in vitro. Results are expressed as mean ± SD, (n=6), * *P* < 0.05.

Further investigation on the encapsulated CHOK1 cells was conducted using MTT test. This test is known as a sensitive and reliable method for evaluating the cellular metabolic activity.^28^ Immobilization of living cells in microcapsules could extend their proliferation time, as according to the CHOK_1_ cells growth curve (Figure 6), the cell numbers dropped from seventh day, while as observed in Figure 7, the cellular metabolic activity inside both cALA and cAPA grew gradually within four weeks. This finding is in line with the other studies reporting extended proliferation rate for different kinds of encapsulated cell lines.^10,21,27,29^ However, the OD values were different between cALA and cAPA, which may be due to the initial less post encapsulation cell survival in cAPA in comparison with cALA.

## Conclusion

After evaluating the empty ALA and APA microcapsules, here in this study, the fate of CHOK1 cells inside these microcapsules (as the most important concern) was compared. CHOK_1_ cells were immobilized inside the ALA and APA microcapsules using electrostatic bead generator, with the approximate diameter of 450 µm. The incorporation of live cells inside the microcapsules did not change the mechanical stability of the microcapsules since they showed a similar pattern of size growth and similar mechanical strength to the empty ALA and APA microcapsules. The comparison of cell viability as well as metabolic activity inside the cALA and cAPA was highly in favor of cALA, which could be due to the significant higher cell survival post encapsulation in cALA in comparison with cAPA. Here, based on the higher cell viability and cellular metabolic activity inside the cALA in comparison with cAPA, it could be concluded that LPEI offered better properties as a covering layer for encapsulation of live cells; therefore it could be used as a proper cost-effective alternative to PLL in cell microencapsulation.

## Ethical Issues

Not applicable.

## Conflict of Interest

Authors declare no conflict of interest in this study.

## Acknowledgments

The authors wish to thank Mr. Farhad Rafraf and Mr. S. Mahyar Foroutanfar for their valuable efforts in design of encapsulator device and the graphical parts of this project. This study was supported by Shahid Beheshti University of Medical Sciences (SBMU) and Pasteur Institute of Iran (IPI).
